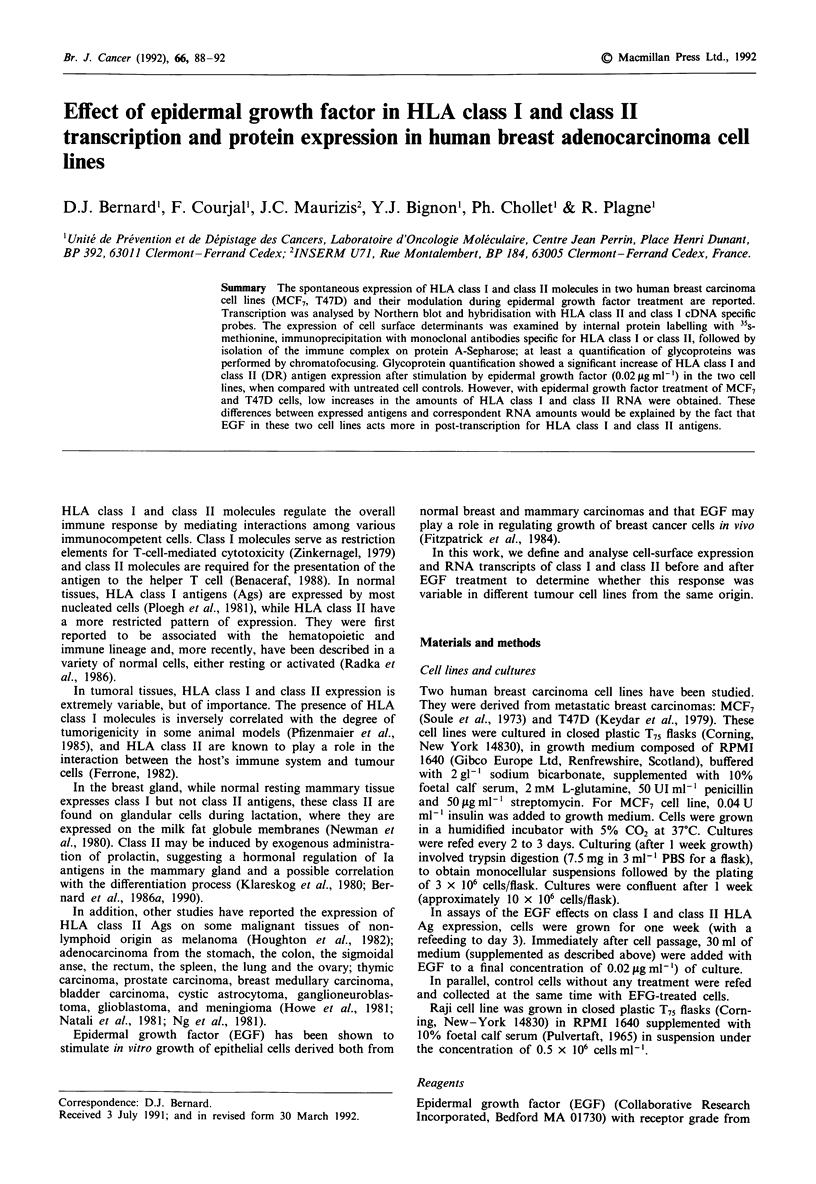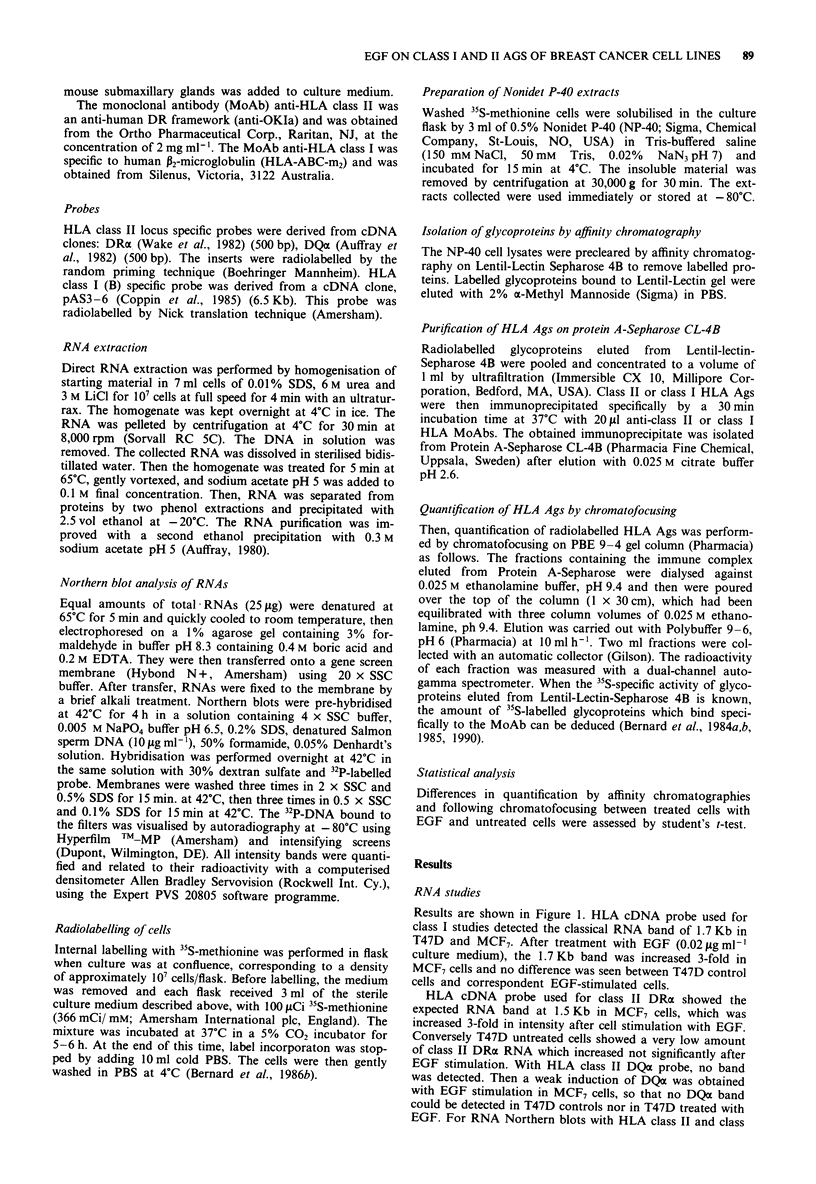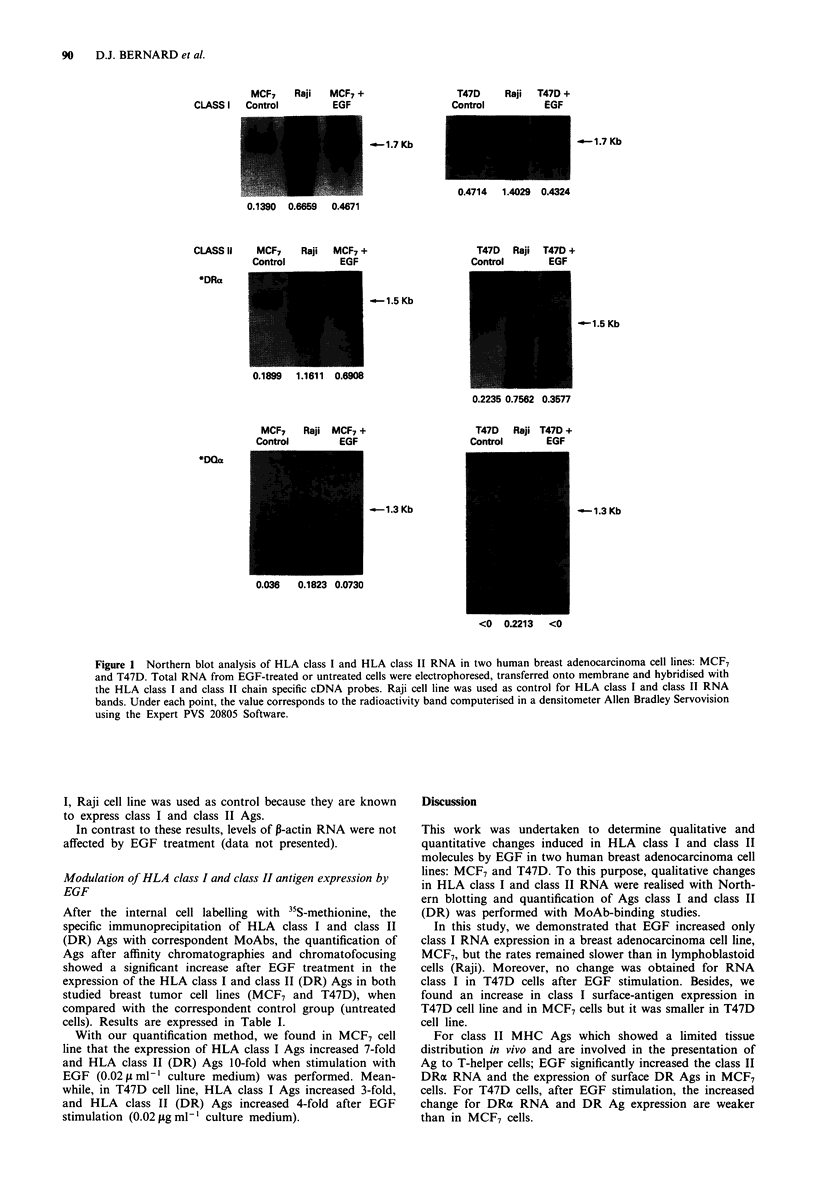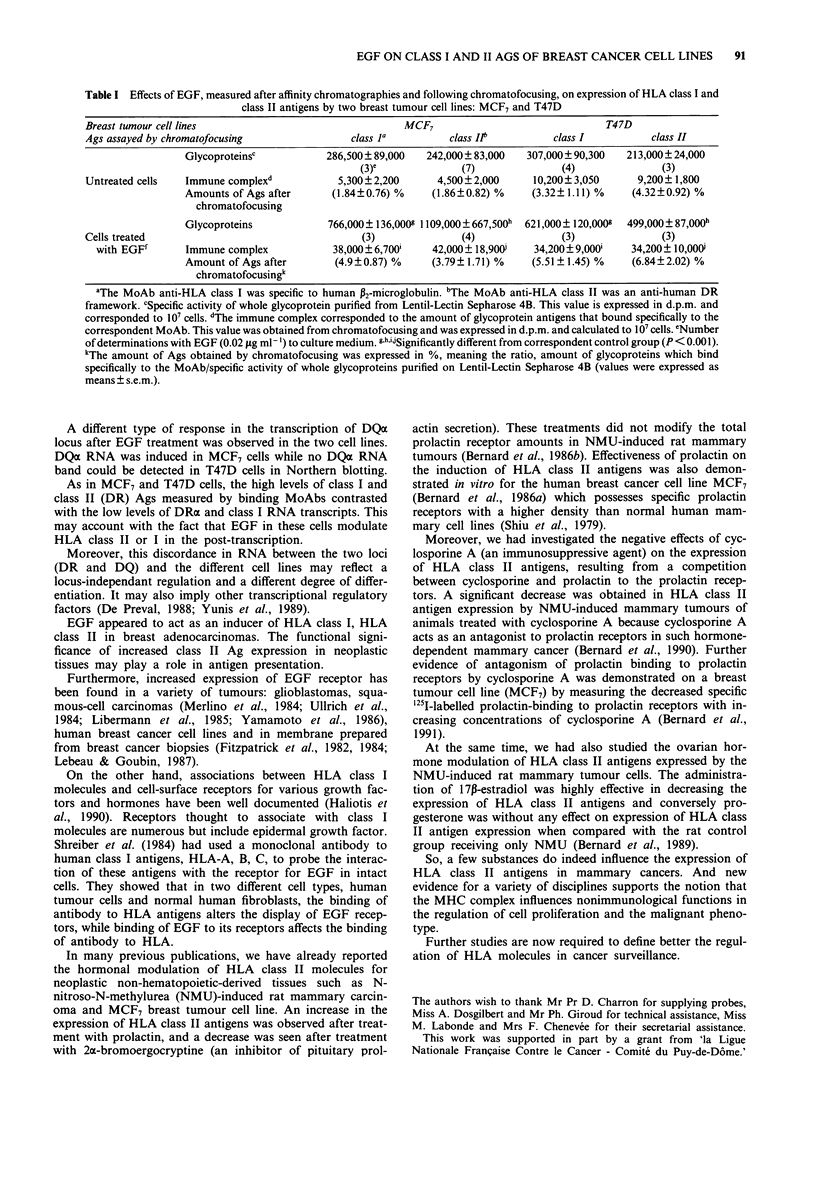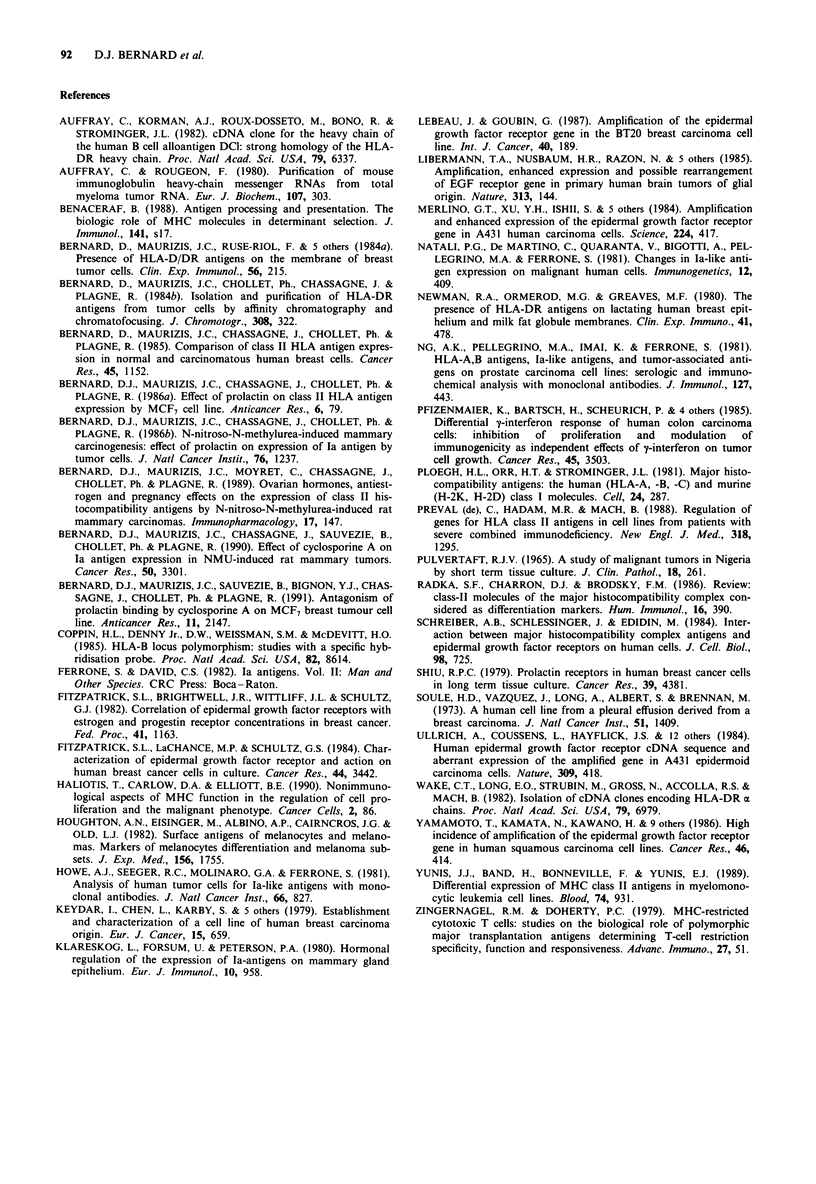# Effect of epidermal growth factor in HLA class I and class II transcription and protein expression in human breast adenocarcinoma cell lines.

**DOI:** 10.1038/bjc.1992.222

**Published:** 1992-07

**Authors:** D. J. Bernard, F. Courjal, J. C. Maurizis, Y. J. Bignon, P. Chollet, R. Plagne

**Affiliations:** Unité de Prévention et de Dépistage des Cancers, Centre Jean Perrin, Clermont-Ferrand, France.

## Abstract

**Images:**


*Br. J. Cancer* (1992), **66**, 88–92 © Macmillan Press Ltd., 1992

### Effect of epidermal growth factor in HLA class I and class II transcription and protein expression in human breast adenocarcinoma cell lines

D.J. Bernard[1], F. Courjal[1], J.C. Maurizis[2], Y.J. Bignon[1], Ph. Chollet[1] & R. Plagne[1]

[1]*Unité de Prévention et de Dépistage des Cancers, Laboratoire d'Oncologie Moléculaire, Centre Jean Perrin, Place Henri Dunant, BP 392, 63011 Clermont-Ferrand Cedex;* [2]*INSERM U71, Rue Montalembert, BP 184, 63005 Clermont-Ferrand Cedex, France.*

**Summary** The spontaneous expression of HLA class I and class II molecules in two human breast carcinoma cell lines ($MCF_7$, T47D) and their modulation during epidermal growth factor treatment are reported. Transcription was analysed by Northern blot and hybridisation with HLA class II and class I cDNA specific probes. The expression of cell surface determinants was examined by internal protein labelling with $^{35}$s-methionine, immunoprecipitation with monoclonal antibodies specific for HLA class I or class II, followed by isolation of the immune complex on protein A-Sepharose; at least a quantification of glycoproteins was performed by chromatofocusing. Glycoprotein quantification showed a significant increase of HLA class I and class II (DR) antigen expression after stimulation by epidermal growth factor ($0.02\ \mu g\ ml^{-1}$) in the two cell lines, when compared with untreated cell controls. However, with epidermal growth factor treatment of $MCF_7$ and T47D cells, low increases in the amounts of HLA class I and class II RNA were obtained. These differences between expressed antigens and correspondent RNA amounts would be explained by the fact that EGF in these two cell lines acts more in post-transcription for HLA class I and class II antigens.

HLA class I and class II molecules regulate the overall immune response by mediating interactions among various immunocompetent cells. Class I molecules serve as restriction elements for T-cell-mediated cytotoxicity (Zinkernagel, 1979) and class II molecules are required for the presentation of the antigen to the helper T cell (Benaceraf, 1988). In normal tissues, HLA class I antigens (Ags) are expressed by most nucleated cells (Ploegh *et al.*, 1981), while HLA class II have a more restricted pattern of expression. They were first reported to be associated with the hematopoietic and immune lineage and, more recently, have been described in a variety of normal cells, either resting or activated (Radka *et al.*, 1986).

In tumoral tissues, HLA class I and class II expression is extremely variable, but of importance. The presence of HLA class I molecules is inversely correlated with the degree of tumorigenicity in some animal models (Pfizenmaier *et al.*, 1985), and HLA class II are known to play a role in the interaction between the host's immune system and tumour cells (Ferrone, 1982).

In the breast gland, while normal resting mammary tissue expresses class I but not class II antigens, these class II are found on glandular cells during lactation, where they are expressed on the milk fat globule membranes (Newman *et al.*, 1980). Class II may be induced by exogenous administration of prolactin, suggesting a hormonal regulation of Ia antigens in the mammary gland and a possible correlation with the differentiation process (Klareskog *et al.*, 1980; Bernard *et al.*, 1986*a*, 1990).

In addition, other studies have reported the expression of HLA class II Ags on some malignant tissues of non-lymphoid origin as melanoma (Houghton *et al.*, 1982); adenocarcinoma from the stomach, the colon, the sigmoidal anse, the rectum, the spleen, the lung and the ovary; thymic carcinoma, prostate carcinoma, breast medullary carcinoma, bladder carcinoma, cystic astrocytoma, ganglioneuroblastoma, glioblastoma, and meningioma (Howe *et al.*, 1981; Natali *et al.*, 1981; Ng *et al.*, 1981).

Epidermal growth factor (EGF) has been shown to stimulate *in vitro* growth of epithelial cells derived both from normal breast and mammary carcinomas and that EGF may play a role in regulating growth of breast cancer cells *in vivo* (Fitzpatrick *et al.*, 1984).

In this work, we define and analyse cell-surface expression and RNA transcripts of class I and class II before and after EGF treatment to determine whether this response was variable in different tumour cell lines from the same origin.

### Materials and methods

### *Cell lines and cultures*

Two human breast carcinoma cell lines have been studied. They were derived from metastatic breast carcinomas: $MCF_7$ (Soule *et al.*, 1973) and T47D (Keydar *et al.*, 1979). These cell lines were cultured in closed plastic $T_{75}$ flasks (Corning, New York 14830), in growth medium composed of RPMI 1640 (Gibco Europe Ltd, Renfrewshire, Scotland), buffered with $2\ g l^{-1}$ sodium bicarbonate, supplemented with 10% foetal calf serum, 2 mM L-glutamine, $50\ UI\ ml^{-1}$ penicillin and $50\ \mu g\ ml^{-1}$ streptomycin. For $MCF_7$ cell line, $0.04\ U\ ml^{-1}$ insulin was added to growth medium. Cells were grown in a humidified incubator with 5% $CO_2$ at 37°C. Cultures were refed every 2 to 3 days. Culturing (after 1 week growth) involved trypsin digestion (7.5 mg in $3\ ml^{-1}$ PBS for a flask), to obtain monocellular suspensions followed by the plating of $3 \times 10^6$ cells/flask. Cultures were confluent after 1 week (approximately $10 \times 10^6$ cells/flask).

In assays of the EGF effects on class I and class II HLA Ag expression, cells were grown for one week (with a refeeding to day 3). Immediately after cell passage, 30 ml of medium (supplemented as described above) were added with EGF to a final concentration of $0.02\ \mu g\ ml^{-1}$) of culture.

In parallel, control cells without any treatment were refed and collected at the same time with EFG-treated cells.

Raji cell line was grown in closed plastic $T_{75}$ flasks (Corning, New-York 14830) in RPMI 1640 supplemented with 10% foetal calf serum (Pulvertaft, 1965) in suspension under the concentration of $0.5 \times 10^6$ cells $ml^{-1}$.

### *Reagents*

Epidermal growth factor (EGF) (Collaborative Research Incorporated, Bedford MA 01730) with receptor grade from

Correspondence: D.J. Bernard.
Received 3 July 1991; and in revised form 30 March 1992.

mouse submaxillary glands was added to culture medium.

The monoclonal antibody (MoAb) anti-HLA class II was an anti-human DR framework (anti-OKIa) and was obtained from the Ortho Pharmaceutical Corp., Raritan, NJ, at the concentration of 2 mg ml$^{-1}$. The MoAb anti-HLA class I was specific to human $\beta_2$-microglobulin (HLA-ABC-m$_2$) and was obtained from Silenus, Victoria, 3122 Australia.

*Probes*

HLA class II locus specific probes were derived from cDNA clones: DRα (Wake *et al.*, 1982) (500 bp), DQα (Auffray *et al.*, 1982) (500 bp). The inserts were radiolabelled by the random priming technique (Boehringer Mannheim). HLA class I (B) specific probe was derived from a cDNA clone, pAS3-6 (Coppin *et al.*, 1985) (6.5 Kb). This probe was radiolabelled by Nick translation technique (Amersham).

*RNA extraction*

Direct RNA extraction was performed by homogenisation of starting material in 7 ml cells of 0.01% SDS, 6 M urea and 3 M LiCl for $10^7$ cells at full speed for 4 min with an ultraturrax. The homogenate was kept overnight at 4°C in ice. The RNA was pelleted by centrifugation at 4°C for 30 min at 8,000 rpm (Sorvall RC 5C). The DNA in solution was removed. The collected RNA was dissolved in sterilised bidistillated water. Then the homogenate was treated for 5 min at 65°C, gently vortexed, and sodium acetate pH 5 was added to 0.1 M final concentration. Then, RNA was separated from proteins by two phenol extractions and precipitated with 2.5 vol ethanol at −20°C. The RNA purification was improved with a second ethanol precipitation with 0.3 M sodium acetate pH 5 (Auffray, 1980).

*Northern blot analysis of RNAs*

Equal amounts of total·RNAs (25 µg) were denatured at 65°C for 5 min and quickly cooled to room temperature, then electrophoresed on a 1% agarose gel containing 3% formaldehyde in buffer pH 8.3 containing 0.4 M boric acid and 0.2 M EDTA. They were then transferred onto a gene screen membrane (Hybond N+, Amersham) using 20 × SSC buffer. After transfer, RNAs were fixed to the membrane by a brief alkali treatment. Northern blots were pre-hybridised at 42°C for 4 h in a solution containing 4 × SSC buffer, 0.005 M $NaPO_4$ buffer pH 6.5, 0.2% SDS, denatured Salmon sperm DNA (10 µg ml$^{-1}$), 50% formamide, 0.05% Denhardt's solution. Hybridisation was performed overnight at 42°C in the same solution with 30% dextran sulfate and $^{32}$P-labelled probe. Membranes were washed three times in 2 × SSC and 0.5% SDS for 15 min. at 42°C, then three times in 0.5 × SSC and 0.1% SDS for 15 min at 42°C. The $^{32}$P-DNA bound to the filters was visualised by autoradiography at −80°C using Hyperfilm ™-MP (Amersham) and intensifying screens (Dupont, Wilmington, DE). All intensity bands were quantified and related to their radioactivity with a computerised densitometer Allen Bradley Servovision (Rockwell Int. Cy.), using the Expert PVS 20805 software programme.

*Radiolabelling of cells*

Internal labelling with $^{35}$S-methionine was performed in flask when culture was at confluence, corresponding to a density of approximately $10^7$ cells/flask. Before labelling, the medium was removed and each flask received 3 ml of the sterile culture medium described above, with 100 µCi $^{35}$S-methionine (366 mCi/ mM; Amersham International plc, England). The mixture was incubated at 37°C in a 5% $CO_2$ incubator for 5–6 h. At the end of this time, label incorporaton was stopped by adding 10 ml cold PBS. The cells were then gently washed in PBS at 4°C (Bernard *et al.*, 1986*b*).

*Preparation of Nonidet P-40 extracts*

Washed $^{35}$S-methionine cells were solubilised in the culture flask by 3 ml of 0.5% Nonidet P-40 (NP-40; Sigma, Chemical Company, St-Louis, NO, USA) in Tris-buffered saline (150 mM NaCl, 50 mM Tris, 0.02% $NaN_3$ pH 7) and incubated for 15 min at 4°C. The insoluble material was removed by centrifugation at 30,000 g for 30 min. The extracts collected were used immediately or stored at −80°C.

*Isolation of glycoproteins by affinity chromatography*

The NP-40 cell lysates were precleared by affinity chromatography on Lentil-Lectin Sepharose 4B to remove labelled proteins. Labelled glycoproteins bound to Lentil-Lectin gel were eluted with 2% α-Methyl Mannoside (Sigma) in PBS.

*Purification of HLA Ags on protein A-Sepharose CL-4B*

Radiolabelled glycoproteins eluted from Lentil-lectin-Sepharose 4B were pooled and concentrated to a volume of 1 ml by ultrafiltration (Immersible CX 10, Millipore Corporation, Bedford, MA, USA). Class II or class I HLA Ags were then immunoprecipitated specifically by a 30 min incubation time at 37°C with 20 µl anti-class II or class I HLA MoAbs. The obtained immunoprecipitate was isolated from Protein A-Sepharose CL-4B (Pharmacia Fine Chemical, Uppsala, Sweden) after elution with 0.025 M citrate buffer pH 2.6.

*Quantification of HLA Ags by chromatofocusing*

Then, quantification of radiolabelled HLA Ags was performed by chromatofocusing on PBE 9-4 gel column (Pharmacia) as follows. The fractions containing the immune complex eluted from Protein A-Sepharose were dialysed against 0.025 M ethanolamine buffer, pH 9.4 and then were poured over the top of the column (1 × 30 cm), which had been equilibrated with three column volumes of 0.025 M ethanolamine, ph 9.4. Elution was carried out with Polybuffer 9-6, pH 6 (Pharmacia) at 10 ml h$^{-1}$. Two ml fractions were collected with an automatic collector (Gilson). The radioactivity of each fraction was measured with a dual-channel autogamma spectrometer. When the $^{35}$S-specific activity of glycoproteins eluted from Lentil-Lectin-Sepharose 4B is known, the amount of $^{35}$S-labelled glycoproteins which bind specifically to the MoAb can be deduced (Bernard *et al.*, 1984*a*,*b*, 1985, 1990).

*Statistical analysis*

Differences in quantification by affinity chromatographies and following chromatofocusing between treated cells with EGF and untreated cells were assessed by student's *t*-test.

### Results

*RNA studies*

Results are shown in Figure 1. HLA cDNA probe used for class I studies detected the classical RNA band of 1.7 Kb in T47D and $MCF_7$. After treatment with EGF (0.02 µg ml$^{-1}$ culture medium), the 1.7 Kb band was increased 3-fold in $MCF_7$ cells and no difference was seen between T47D control cells and correspondent EGF-stimulated cells.

HLA cDNA probe used for class II DRα showed the expected RNA band at 1.5 Kb in $MCF_7$ cells, which was increased 3-fold in intensity after cell stimulation with EGF. Conversely T47D untreated cells showed a very low amount of class II DRα RNA which increased not significantly after EGF stimulation. With HLA class II DQα probe, no band was detected. Then a weak induction of DQα was obtained with EGF stimulation in $MCF_7$ cells, so that no DQα band could be detected in T47D controls nor in T47D treated with EGF. For RNA Northern blots with HLA class II and class

**Figure 1** Northern blot analysis of HLA class I and HLA class II RNA in two human breast adenocarcinoma cell lines: $MCF_7$ and T47D. Total RNA from EGF-treated or untreated cells were electrophoresed, transferred onto membrane and hybridised with the HLA class I and class II chain specific cDNA probes. Raji cell line was used as control for HLA class I and class II RNA bands. Under each point, the value corresponds to the radioactivity band computerised in a densitometer Allen Bradley Servovision using the Expert PVS 20805 Software.

I, Raji cell line was used as control because they are known to express class I and class II Ags.

In contrast to these results, levels of β-actin RNA were not affected by EGF treatment (data not presented).

*Modulation of HLA class I and class II antigen expression by EGF*

After the internal cell labelling with $^{35}$S-methionine, the specific immunoprecipitation of HLA class I and class II (DR) Ags with correspondent MoAbs, the quantification of Ags after affinity chromatographies and chromatofocusing showed a significant increase after EGF treatment in the expression of the HLA class I and class II (DR) Ags in both studied breast tumor cell lines ($MCF_7$ and T47D), when compared with the correspondent control group (untreated cells). Results are expressed in Table I.

With our quantification method, we found in $MCF_7$ cell line that the expression of HLA class I Ags increased 7-fold and HLA class II (DR) Ags 10-fold when stimulation with EGF ($0.02\,\mu\,ml^{-1}$ culture medium) was performed. Meanwhile, in T47D cell line, HLA class I Ags increased 3-fold, and HLA class II (DR) Ags increased 4-fold after EGF stimulation ($0.02\,\mu g\,ml^{-1}$ culture medium).

### Discussion

This work was undertaken to determine qualitative and quantitative changes induced in HLA class I and class II molecules by EGF in two human breast adenocarcinoma cell lines: $MCF_7$ and T47D. To this purpose, qualitative changes in HLA class I and class II RNA were realised with Northern blotting and quantification of Ags class I and class II (DR) was performed with MoAb-binding studies.

In this study, we demonstrated that EGF increased only class I RNA expression in a breast adenocarcinoma cell line, $MCF_7$, but the rates remained slower than in lymphoblastoid cells (Raji). Moreover, no change was obtained for RNA class I in T47D cells after EGF stimulation. Besides, we found an increase in class I surface-antigen expression in T47D cell line and in $MCF_7$ cells but it was smaller in T47D cell line.

For class II MHC Ags which showed a limited tissue distribution *in vivo* and are involved in the presentation of Ag to T-helper cells; EGF significantly increased the class II DRα RNA and the expression of surface DR Ags in $MCF_7$ cells. For T47D cells, after EGF stimulation, the increased change for DRα RNA and DR Ag expression are weaker than in $MCF_7$ cells.

**Table I** Effects of EGF, measured after affinity chromatographies and following chromatofocusing, on expression of HLA class I and class II antigens by two breast tumour cell lines: $MCF_7$ and T47D

| *Breast tumour cell lines* | | *$MCF_7$* | | *T47D* | |
|---|---|---|---|---|---|
| *Ags assayed by chromatofocusing* | | *class I*[a] | *class II*[b] | *class I* | *class II* |
| | Glycoproteins[c] | 286,500 ± 89,000 (3)[e] | 242,000 ± 83,000 (7) | 307,000 ± 90,300 (4) | 213,000 ± 24,000 (3) |
| Untreated cells | Immune complex[d] | 5,300 ± 2,200 | 4,500 ± 2,000 | 10,200 ± 3,050 | 9,200 ± 1,800 |
| | Amounts of Ags after chromatofocusing | (1.84 ± 0.76) % | (1.86 ± 0.82) % | (3.32 ± 1.11) % | (4.32 ± 0.92) % |
| | Glycoproteins | 766,000 ± 136,000[g] (3) | 1109,000 ± 667,500[h] (4) | 621,000 ± 120,000[g] (3) | 499,000 ± 87,000[h] (3) |
| Cells treated with EGF[f] | Immune complex | 38,000 ± 6,700[i] | 42,000 ± 18,900[j] | 34,200 ± 9,000[i] | 34,200 ± 10,000[j] |
| | Amount of Ags after chromatofocusing[k] | (4.9 ± 0.87) % | (3.79 ± 1.71) % | (5.51 ± 1.45) % | (6.84 ± 2.02) % |

[a]The MoAb anti-HLA class I was specific to human $\beta_2$-microglobulin. [b]The MoAb anti-HLA class II was an anti-human DR framework. [c]Specific activity of whole glycoprotein purified from Lentil-Lectin Sepharose 4B. This value is expressed in d.p.m. and corresponded to $10^7$ cells. [d]The immune complex corresponded to the amount of glycoprotein antigens that bound specifically to the correspondent MoAb. This value was obtained from chromatofocusing and was expressed in d.p.m. and calculated to $10^7$ cells. [e]Number of determinations with EGF (0.02 $\mu g\ ml^{-1}$) to culture medium. [g,h,i,j]Significantly different from correspondent control group ($P < 0.001$). [k]The amount of Ags obtained by chromatofocusing was expressed in %, meaning the ratio, amount of glycoproteins which bind specifically to the MoAb/specific activity of whole glycoproteins purified on Lentil-Lectin Sepharose 4B (values were expressed as means ± s.e.m.).

A different type of response in the transcription of DQα locus after EGF treatment was observed in the two cell lines. DQα RNA was induced in $MCF_7$ cells while no DQα RNA band could be detected in T47D cells in Northern blotting.

As in $MCF_7$ and T47D cells, the high levels of class I and class II (DR) Ags measured by binding MoAbs contrasted with the low levels of DRα and class I RNA transcripts. This may account with the fact that EGF in these cells modulate HLA class II or I in the post-transcription.

Moreover, this discordance in RNA between the two loci (DR and DQ) and the different cell lines may reflect a locus-independant regulation and a different degree of differentiation. It may also imply other transcriptional regulatory factors (De Preval, 1988; Yunis *et al.*, 1989).

EGF appeared to act as an inducer of HLA class I, HLA class II in breast adenocarcinomas. The functional significance of increased class II Ag expression in neoplastic tissues may play a role in antigen presentation.

Furthermore, increased expression of EGF receptor has been found in a variety of tumours: glioblastomas, squamous-cell carcinomas (Merlino *et al.*, 1984; Ullrich *et al.*, 1984; Libermann *et al.*, 1985; Yamamoto *et al.*, 1986), human breast cancer cell lines and in membrane prepared from breast cancer biopsies (Fitzpatrick *et al.*, 1982, 1984; Lebeau & Goubin, 1987).

On the other hand, associations between HLA class I molecules and cell-surface receptors for various growth factors and hormones have been well documented (Haliotis *et al.*, 1990). Receptors thought to associate with class I molecules are numerous but include epidermal growth factor. Shreiber *et al.* (1984) had used a monoclonal antibody to human class I antigens, HLA-A, B, C, to probe the interaction of these antigens with the receptor for EGF in intact cells. They showed that in two different cell types, human tumour cells and normal human fibroblasts, the binding of antibody to HLA antigens alters the display of EGF receptors, while binding of EGF to its receptors affects the binding of antibody to HLA.

In many previous publications, we have already reported the hormonal modulation of HLA class II molecules for neoplastic non-hematopoietic-derived tissues such as N-nitroso-N-methylurea (NMU)-induced rat mammary carcinoma and $MCF_7$ breast tumour cell line. An increase in the expression of HLA class II antigens was observed after treatment with prolactin, and a decrease was seen after treatment with 2α-bromoergocryptine (an inhibitor of pituitary prolactin secretion). These treatments did not modify the total prolactin receptor amounts in NMU-induced rat mammary tumours (Bernard *et al.*, 1986*b*). Effectiveness of prolactin on the induction of HLA class II antigens was also demonstrated *in vitro* for the human breast cancer cell line $MCF_7$ (Bernard *et al.*, 1986*a*) which possesses specific prolactin receptors with a higher density than normal human mammary cell lines (Shiu *et al.*, 1979).

Moreover, we had investigated the negative effects of cyclosporine A (an immunosuppressive agent) on the expression of HLA class II antigens, resulting from a competition between cyclosporine and prolactin to the prolactin receptors. A significant decrease was obtained in HLA class II antigen expression by NMU-induced mammary tumours of animals treated with cyclosporine A because cyclosporine A acts as an antagonist to prolactin receptors in such hormone-dependent mammary cancer (Bernard *et al.*, 1990). Further evidence of antagonism of prolactin binding to prolactin receptors by cyclosporine A was demonstrated on a breast tumour cell line ($MCF_7$) by measuring the decreased specific $^{125}I$-labelled prolactin-binding to prolactin receptors with increasing concentrations of cyclosporine A (Bernard *et al.*, 1991).

At the same time, we had also studied the ovarian hormone modulation of HLA class II antigens expressed by the NMU-induced rat mammary tumour cells. The administration of 17β-estradiol was highly effective in decreasing the expression of HLA class II antigens and conversely progesterone was without any effect on expression of HLA class II antigen expression when compared with the rat control group receiving only NMU (Bernard *et al.*, 1989).

So, a few substances do indeed influence the expression of HLA class II antigens in mammary cancers. And new evidence for a variety of disciplines supports the notion that the MHC complex influences nonimmunological functions in the regulation of cell proliferation and the malignant phenotype.

Further studies are now required to define better the regulation of HLA molecules in cancer surveillance.

The authors wish to thank Mr Pr D. Charron for supplying probes, Miss A. Dosgilbert and Mr Ph. Giroud for technical assistance, Miss M. Labonde and Mrs F. Chenevée for their secretarial assistance.

This work was supported in part by a grant from 'la Ligue Nationale Française Contre le Cancer - Comité du Puy-de-Dôme.'

### References

AUFFRAY, C., KORMAN, A.J., ROUX-DOSSETO, M., BONO, R. & STROMINGER, J.L. (1982). cDNA clone for the heavy chain of the human B cell alloantigen DCl: strong homology of the HLA-DR heavy chain. *Proc. Natl Acad. Sci. USA*, **79**, 6337.

AUFFRAY, C. & ROUGEON, F. (1980). Purification of mouse immunoglobulin heavy-chain messenger RNAs from total myeloma tumor RNA. *Eur. J. Biochem.*, **107**, 303.

BENACERAF, B. (1988). Antigen processing and presentation. The biologic role of MHC molecules in determinant selection. *J. Immunol.*, **141**, s17.

BERNARD, D., MAURIZIS, J.C., RUSE-RIOL, F. & 5 others (1984*a*). Presence of HLA-D/DR antigens on the membrane of breast tumor cells. *Clin. Exp. Immunol.*, **56**, 215.

BERNARD, D., MAURIZIS, J.C., CHOLLET, Ph., CHASSAGNE, J. & PLAGNE, R. (1984*b*). Isolation and purification of HLA-DR antigens from tumor cells by affinity chromatography and chromatofocusing. *J. Chromotogr.*, **308**, 322.

BERNARD, D., MAURIZIS, J.C., CHASSAGNE, J., CHOLLET, Ph. & PLAGNE, R. (1985). Comparison of class II HLA antigen expression in normal and carcinomatous human breast cells. *Cancer Res.*, **45**, 1152.

BERNARD, D.J., MAURIZIS, J.C., CHASSAGNE, J., CHOLLET, Ph. & PLAGNE, R. (1986*a*). Effect of prolactin on class II HLA antigen expression by $MCF_7$ cell line. *Anticancer Res.*, **6**, 79.

BERNARD, D.J., MAURIZIS, J.C., CHASSAGNE, J., CHOLLET, Ph. & PLAGNE, R. (1986*b*). N-nitroso-N-methylurea-induced mammary carcinogenesis: effect of prolactin on expression of Ia antigen by tumor cells. *J. Natl Cancer Instit.*, **76**, 1237.

BERNARD, D.J., MAURIZIS, J.C., MOYRET, C., CHASSAGNE, J., CHOLLET, Ph. & PLAGNE, R. (1989). Ovarian hormones, antiestrogen and pregnancy effects on the expression of class II histocompatibility antigens by N-nitroso-N-methylurea-induced rat mammary carcinomas. *Immunopharmacology*, **17**, 147.

BERNARD, D.J., MAURIZIS, J.C., CHASSAGNE, J., SAUVEZIE, B., CHOLLET, Ph. & PLAGNE, R. (1990). Effect of cyclosporine A on Ia antigen expression in NMU-induced rat mammary tumors. *Cancer Res.*, **50**, 3301.

BERNARD, D.J., MAURIZIS, J.C., SAUVEZIE, B., BIGNON, Y.J., CHASSAGNE, J., CHOLLET, Ph. & PLAGNE, R. (1991). Antagonism of prolactin binding by cyclosporine A on $MCF_7$ breast tumour cell line. *Anticancer Res.*, **11**, 2147.

COPPIN, H.L., DENNY Jr., D.W., WEISSMAN, S.M. & McDEVITT, H.O. (1985). HLA-B locus polymorphism: studies with a specific hybridisation probe. *Proc. Natl Acad. Sci. USA*, **82**, 8614.

FERRONE, S. & DAVID, C.S. (1982). Ia antigens. Vol. II: *Man and Other Species*. CRC Press: Boca-Raton.

FITZPATRICK, S.L., BRIGHTWELL, J.R., WITTLIFF, J.L. & SCHULTZ, G.J. (1982). Correlation of epidermal growth factor receptors with estrogen and progestin receptor concentrations in breast cancer. *Fed. Proc.*, **41**, 1163.

FITZPATRICK, S.L., LaCHANCE, M.P. & SCHULTZ, G.S. (1984). Characterization of epidermal growth factor receptor and action on human breast cancer cells in culture. *Cancer Res.*, **44**, 3442.

HALIOTIS, T., CARLOW, D.A. & ELLIOTT, B.E. (1990). Nonimmunological aspects of MHC function in the regulation of cell proliferation and the malignant phenotype. *Cancer Cells*, **2**, 86.

HOUGHTON, A.N., EISINGER, M., ALBINO, A.P., CAIRNCROS, J.G. & OLD, L.J. (1982). Surface antigens of melanocytes and melanomas. Markers of melanocytes differentiation and melanoma subsets. *J. Exp. Med.*, **156**, 1755.

HOWE, A.J., SEEGER, R.C., MOLINARO, G.A. & FERRONE, S. (1981). Analysis of human tumor cells for Ia-like antigens with monoclonal antibodies. *J. Natl Cancer Inst.*, **66**, 827.

KEYDAR, I., CHEN, L., KARBY, S. & 5 others (1979). Establishment and characterization of a cell line of human breast carcinoma origin. *Eur. J. Cancer*, **15**, 659.

KLARESKOG, L., FORSUM, U. & PETERSON, P.A. (1980). Hormonal regulation of the expression of Ia-antigens on mammary gland epithelium. *Eur. J. Immunol.*, **10**, 958.

LEBEAU, J. & GOUBIN, G. (1987). Amplification of the epidermal growth factor receptor gene in the BT20 breast carcinoma cell line. *Int. J. Cancer*, **40**, 189.

LIBERMANN, T.A., NUSBAUM, H.R., RAZON, N. & 5 others (1985). Amplification, enhanced expression and possible rearrangement of EGF receptor gene in primary human brain tumors of glial origin. *Nature*, **313**, 144.

MERLINO, G.T., XU, Y.H., ISHII, S. & 5 others (1984). Amplification and enhanced expression of the epidermal growth factor receptor gene in A431 human carcinoma cells. *Science*, **224**, 417.

NATALI, P.G., De MARTINO, C., QUARANTA, V., BIGOTTI, A., PELLEGRINO, M.A. & FERRONE, S. (1981). Changes in Ia-like antigen expression on malignant human cells. *Immunogenetics*, **12**, 409.

NEWMAN, R.A., ORMEROD, M.G. & GREAVES, M.F. (1980). The presence of HLA-DR antigens on lactating human breast epithelium and milk fat globule membranes. *Clin. Exp. Immuno.*, **41**, 478.

NG, A.K., PELLEGRINO, M.A., IMAI, K. & FERRONE, S. (1981). HLA-A,B antigens, Ia-like antigens, and tumor-associated antigens on prostate carcinoma cell lines: serologic and immunochemical analysis with monoclonal antibodies. *J. Immunol.*, **127**, 443.

PFIZENMAIER, K., BARTSCH, H., SCHEURICH, P. & 4 others (1985). Differential γ-interferon response of human colon carcinoma cells: inhibition of proliferation and modulation of immunogenicity as independent effects of γ-interferon on tumor cell growth. *Cancer Res.*, **45**, 3503.

PLOEGH, H.L., ORR, H.T. & STROMINGER, J.L. (1981). Major histocompatibility antigens: the human (HLA-A, -B, -C) and murine (H-2K, H-2D) class I molecules. *Cell*, **24**, 287.

PREVAL (de), C., HADAM, M.R. & MACH, B. (1988). Regulation of genes for HLA class II antigens in cell lines from patients with severe combined immunodeficiency. *New Engl. J. Med.*, **318**, 1295.

PULVERTAFT, R.J.V. (1965). A study of malignant tumors in Nigeria by short term tissue culture. *J. Clin. Pathol.*, **18**, 261.

RADKA, S.F., CHARRON, D.J. & BRODSKY, F.M. (1986). Review: class-II molecules of the major histocompatibility complex considered as differentiation markers. *Hum. Immunol.*, **16**, 390.

SCHREIBER, A.B., SCHLESSINGER, J. & EDIDIN, M. (1984). Interaction between major histocompatibility complex antigens and epidermal growth factor receptors on human cells. *J. Cell. Biol.*, **98**, 725.

SHIU, R.P.C. (1979). Prolactin receptors in human breast cancer cells in long term tissue culture. *Cancer Res.*, **39**, 4381.

SOULE, H.D., VAZQUEZ, J., LONG, A., ALBERT, S. & BRENNAN, M. (1973). A human cell line from a pleural effusion derived from a breast carcinoma. *J. Natl Cancer Inst.*, **51**, 1409.

ULLRICH, A., COUSSENS, L., HAYFLICK, J.S. & 12 others (1984). Human epidermal growth factor receptor cDNA sequence and aberrant expression of the amplified gene in A431 epidermoid carcinoma cells. *Nature*, **309**, 418.

WAKE, C.T., LONG, E.O., STRUBIN, M., GROSS, N., ACCOLLA, R.S. & MACH, B. (1982). Isolation of cDNA clones encoding HLA-DR α chains. *Proc. Natl Acad. Sci. USA*, **79**, 6979.

YAMAMOTO, T., KAMATA, N., KAWANO, H. & 9 others (1986). High incidence of amplification of the epidermal growth factor receptor gene in human squamous carcinoma cell lines. *Cancer Res.*, **46**, 414.

YUNIS, J.J., BAND, H., BONNEVILLE, F. & YUNIS, E.J. (1989). Differential expression of MHC class II antigens in myelomonocytic leukemia cell lines. *Blood*, **74**, 931.

ZINGERNAGEL, R.M. & DOHERTY, P.C. (1979). MHC-restricted cytotoxic T cells: studies on the biological role of polymorphic major transplantation antigens determining T-cell restriction specificity, function and responsiveness. *Advanc. Immuno.*, **27**, 51.